# Identification of Chalcone Derivatives as Inhibitors of *Leishmania infantum* Arginase and Promising Antileishmanial Agents

**DOI:** 10.3389/fchem.2020.624678

**Published:** 2021-01-14

**Authors:** Andreza R. Garcia, Danielle M. P. Oliveira, Jessica B. Jesus, Alessandra M. T. Souza, Ana Carolina R. Sodero, Alane B. Vermelho, Ivana C. R. Leal, Rodrigo Octavio M. A. Souza, Leandro S. M. Miranda, Anderson S. Pinheiro, Igor A. Rodrigues

**Affiliations:** ^1^Graduate Program in Pharmaceutical Sciences, School of Pharmacy, Federal University of Rio de Janeiro, Rio de Janeiro, Brazil; ^2^Department of Biochemistry, Institute of Chemistry, Federal University of Rio de Janeiro, Rio de Janeiro, Brazil; ^3^Department of Drugs and Medicines, School of Pharmacy, Federal University of Rio de Janeiro, Rio de Janeiro, Brazil; ^4^Department of General Microbiology, Institute of Microbiology Paulo de Goes, Federal University of Rio de Janeiro, Rio de Janeiro, Brazil; ^5^Department of Natural Products and Food, School of Pharmacy, Federal University of Rio de Janeiro, Rio de Janeiro, Brazil; ^6^Department of Organic Chemistry, Institute of Chemistry, Federal University of Rio de Janeiro, Rio de Janeiro, Brazil

**Keywords:** *Leishmania infantum*, arginase, inhibition, chalcone, antileishmanial activity

## Abstract

Arginase catalyzes the hydrolysis of l-arginine into l-ornithine and urea, acting as a key enzyme in the biosynthesis of polyamines. *Leishmania* growth and survival is dependent on polyamine biosynthesis; therefore, inhibition of *Leishmania* arginase may be a promising therapeutic strategy. Here, we evaluated a series of thirty-six chalcone derivatives as potential inhibitors of *Leishmania infantum* arginase (LiARG). In addition, the activity of selected inhibitors against *L. infantum* parasites was assessed *in vitro*. Seven compounds exhibited LiARG inhibition above 50% at 100 μM. Among them, compounds LC41, LC39, and LC32 displayed the greatest inhibition values (72.3 ± 0.3%, 71.9 ± 11.6%, and 69.5 ± 7.9%, respectively). Molecular docking studies predicted hydrogen bonds and hydrophobic interactions between the most active chalcones (LC32, LC39, and LC41) and specific residues from LiARG's active site, such as His140, Asn153, His155, and Ala193. Compound LC32 showed the highest activity against *L. infantum* promastigotes (IC_50_ of 74.1 ± 10.0 μM), whereas compounds LC39 and LC41 displayed the best results against intracellular amastigotes (IC_50_ of 55.2 ± 3.8 and 70.4 ± 9.6 μM, respectively). Moreover, compound LC39 showed more selectivity against parasites than host cells (macrophages), with a selectivity index (SI) of 107.1, even greater than that of the reference drug Fungizone®. Computational pharmacokinetic and toxicological evaluations showed high oral bioavailability and low toxicity for the most active compounds. The results presented here support the use of substituted chalcone skeletons as promising LiARG inhibitors and antileishmanial drug candidates.

## Introduction

*Leishmania infantum* is the etiological agent of visceral leishmaniasis (VL), a lethal infectious disease that afflicts neglected populations mainly distributed in Africa, Asia and Latin America. Recently, the World Health Organization estimated that 30,000 new cases of VL occur worldly (World Health Organization, 2020). Despite the global effort to develop effective vaccines and new leishmanicidal drugs, the control of this disease remains a challenge, especially as other clinical forms of leishmaniasis have emerged. Among the drug discovery strategies, the search for specific inhibitors of *Leishmania* essential enzymes has shown promising results (das Neves et al., 2019; Reguera et al., 2019).

Arginase plays a pivotal role for the survival of *Leishmania*. This enzyme acts as a binuclear manganese metalloprotease responsible for l-arginine bioconversion into ornithine, which represents the first step of the polyamine pathway. In the past few years, *Leishmania* arginase (ARG) has been proposed as a potential target for new drug candidates of natural (Girard-Thernier et al., 2015; Glisic et al., 2016; da Silva et al., 2019) or synthetic origin (Crizanto de Lima et al., 2019). Indeed, ARG inhibition affects not only the polyamine pathway and, consequently, parasite growth, but also the thiol pathway since spermidine, the final product of the polyamine pathway, is converted into trypanothione. The thiol pathway is essential for the elimination of reactive oxygen species (ROS) generated by the macrophage oxidative burst and establishment of infection (Ilari et al., 2017; Pessenda and da Silva, 2020). Previously, Muleme et al. (2009) demonstrated that *Leishmania major* ARG null mutants showed phenotypes of diminished proliferation *in vitro* (macrophage infection) and *in vivo* (Balb/C mouse infection) contexts; this finding reinforces that ARG is essential for *Leishmania* pathophysiology and is therefore an intriguing target for new therapeutics.

Chalcones, or 1,3-diaryl-2-propen-1-ones, are naturally occurring compounds belonging to the flavonoid family. They are characterized by a simple scaffold of two phenolic rings connected by a three-carbon α,β unsaturated carbonyl bridge (Rosa et al., 2019). Compounds with chalcone structures exhibit diverse biological activities and, consequently, they have attracted researchers' attention as a curious starting point for the synthesis of molecular entities with potential pharmacological properties (Gomes et al., 2017b). Indeed, antitumor, anti-inflammatory, antioxidant (Venturelli et al., 2016), and antimicrobial (Noreljaleel et al., 2018) activities have been described for natural chalcones (and derivatives thereof). Moreover, chalcone-based compounds exhibited inhibitory effects against trypanosomatids, including *Trypanosoma cruzi* and *Leishmania* (Espinoza-Hicks et al., 2019).

Previously, our group described the inhibition of *L. infantum* recombinant arginase (LiARG) by naturally occurring phenolic substances (Garcia et al., 2019). Here, we report on a new possible mechanism of action of chalcone derivatives against *L. infantum* by showing that these chemical entities inhibit LiARG. We screened a library of thirty-six synthetic chalcone derivatives against LiARG. We showed by molecular docking that the most active inhibitors interact directly with essential residues at the enzyme's active site. Moreover, we investigated the antileishmanial effect of chalcone derivatives against the promastigote and intracellular amastigote forms of *L. infantum*, as well as their cytotoxicity, in order to determine their selectivity. Furthermore, preliminary pharmacokinetic and toxicological evaluations were performed using *in silico* approach. Finally, we evaluated the ability of chalcone derivatives to modulate the host immune response.

## Materials and Methods

### *Leishmania infantum* Arginase (LiARG) Expression and Purification

LiARG expression and purification was performed as described previously (Garcia et al., 2019). *Escherichia coli* BL21 (DE3) cells were transformed with RP1B-LiARG. Cells were grown at 37°C until mid-exponential phase (Abs_600_ ~ 0.6). Protein expression was induced with 1 mM IPTG and cells were grown for 16 h at 30°C. Cells were harvested by centrifugation, resuspended in lysis buffer [50 mM Tris-HCl (pH 8.0), 500 mM NaCl, 5 mM imidazol, 0.1% Triton-X 100, 250 μM PMSF, 10 mM β-mercaptoethanol] and lysed by sonication (15 cycles 60 s on and 60 s off, 100 W). The cell lysate was centrifuged (8,000 X *g*, 40 min, 4°C), and the clarified supernatant was loaded onto a His-Trap HP column (GE Healthcare, USA). A wash step with 100 mM manganese chloride was carried out for enzyme activation. LiARG was eluted with an imidazole gradient ranging from 5 to 500 mM. Fractions containing LiARG were pooled and dialyzed in the presence of His_6_-TEV protease for removal of the N-terminal His_6_ tag. A second nickel-affinity chromatography step was used to remove the His_6_ tag and His_6_-TEV. Finally, purified LiARG was dialyzed against [50 mM CHES (pH 9.5), 100 mM NaCl, 5 mM DTT, 250 μM PMSF], concentrated and stored at −80°C.

### Enzyme Inhibition Assay

**Figure 1:**
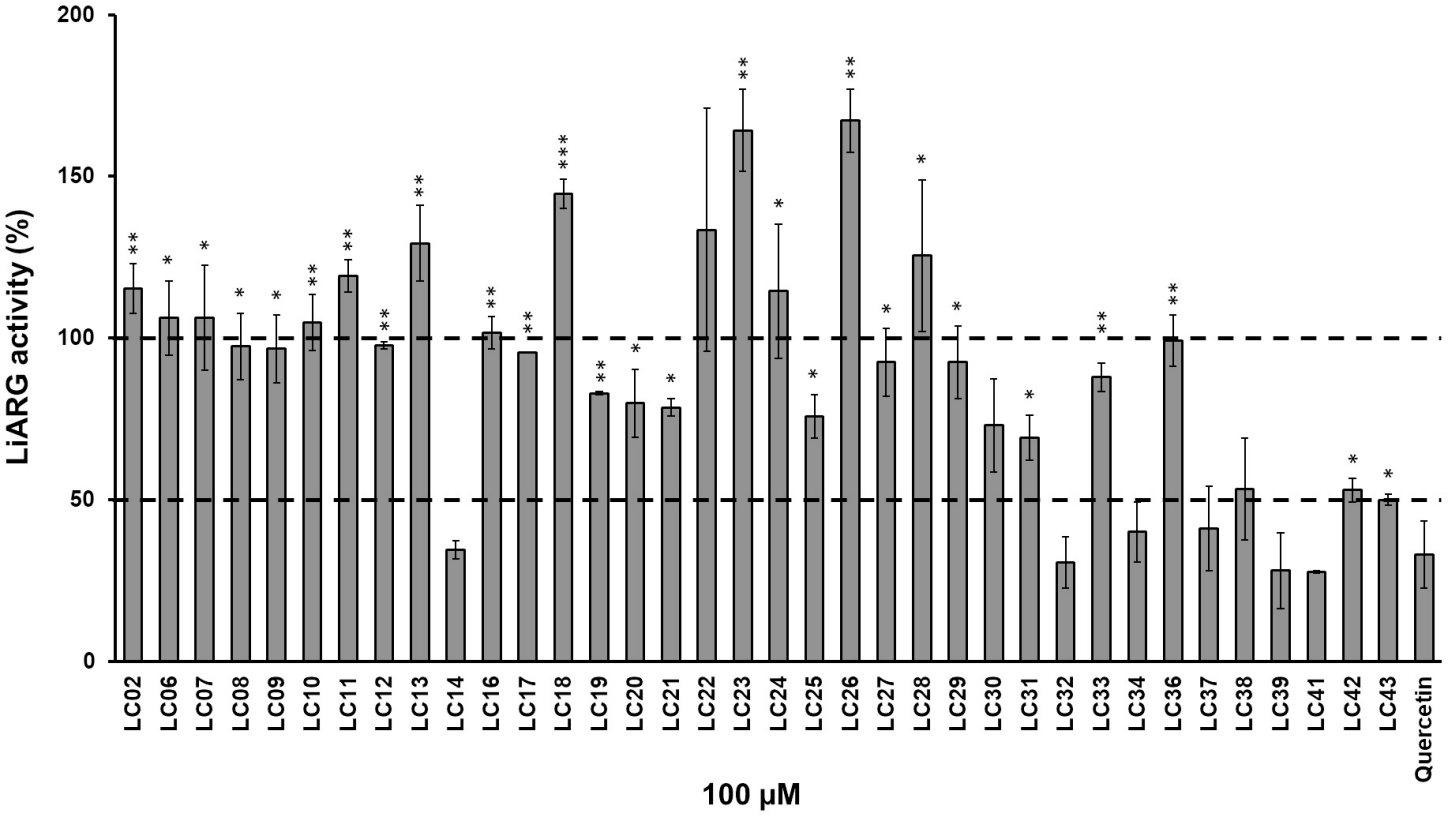
Inhibition of LiARG by chalcone derivatives. LiARG ability to catalyze the conversion of l-arginine into urea was measured in the presence of thirty-six different chalcone derivatives at 100 μM concentration. Quercetin (100 μM) was used as a reference LiARG inhibitor. The control reaction was performed in the absence of any inhibitor. The results are expressed as percentage of LiARG activity taking the enzymatic activity of the control as 100%. A statistical analysis using Student's *t*-test was performed comparing each chalcone derivative to quercetin (control). *P*-values < 0.05 (one asterisk), <0.005 (two asterisks) and <0.0005 (three asterisks) were considered significantly different from the control. The dashed lines represent 100% (control) and 50% LiARG activity, which is the cutoff used for selection of the best inhibitors.

LiARG activity was measured using the UREA CE kit (Labtest, Brazil). Urea concentration was determined spectrophotometrically (SpectraMax M5, Molecular Devices, CA) by hydrolyzing urea into ammonia and then converting ammonia into indophenol blue, which absorbs light at 600 nm (Fawcet and Scott, 1960). Enzyme activity was measured with 0.2 μg/mL LiARG incubated with 50 mM l-arginine in 50 mM CHES (pH 9.5) at 37°C for 5 min. A set of thirty-six synthetic substituted chalcones was used to screen for potential LiARG inhibitors. Synthesis of chalcone derivatives were described previously by Ventura et al. (2015). In addition, the purity of the chalcone derivatives was determined by 200 MHz ^1^H- and 50 MHz ^13^C-NMR as well as mass spectrometry (Ventura et al., 2015). For inhibitor screening, the concentration of chalcone derivatives was kept fixed at 100 μM. Quercetin (100 μM) was used as a reference arginase inhibitor (da Silva et al., 2012). Inhibitors were first dissolved in DMSO at 50 mM. Percentages of inhibition were calculated considering enzyme activity of the control (absence of inhibitor) as 100%. Control reactions were performed in the presence of the same amount of DMSO (0.1%). All measurements were performed in triplicates, at least twice independently. The chalcone derivatives that showed inhibitory activity against LiARG > 50% were selected for the biological assays (Figure 1).

### Molecular Docking

To perform the molecular docking of the selected chalcone derivatives (LC32, LC39, and LC41) into the active site of LiARG, we used LiARG three-dimensional model and docking parameters from our previous work (Garcia et al., 2019). The three-dimensional structures of compounds LC32, LC39, and LC41 were constructed using the Spartan'10 software (Wavefunction Inc, CA). The enzyme:inhibitor complexes generated by molecular docking were analyzed by AutoDockTools 1.5.6 (Morris et al., 2009) and PyMOL (The PyMOL Molecular Graphics System, Version 1.4.1 Schrödinger, LLC.).

### Computational Physicochemical Pharmacokinetic and Toxicological Predictions

The two-dimensional structures were drawn with ACD/ChemSketch (Osterberg and Norinder, 2001). Pharmacokinetic properties and toxicological endpoints were evaluated using qualitative and quantitative models implemented in ADMET Predictor^TM^ software version 9.5 (Simulations Plus, Inc., Lancaster, CA, USA) (Tiwari et al., 2018; Abreu et al., 2020). Using this software, we evaluated the theoretical oral bioavailability based on the Lipinski “rule of five” (Lipinski, 2004). In addition, the metabolic profile of the most active chalcone derivatives (LC32, LC39, and LC41) was evaluated by predicting if the derivatives could act as substrates or inhibitors of nine cytochrome P450 isoforms (CYP1A2, 2A6, 2B6, 2C8, 2C9, 2C19, 2D6, 2E1, and 3A4). The toxicological endpoints evaluated were hepatotoxicity, mutagenicity, carcinogenicity, acute toxicity, and cardiotoxicity (Pinheiro et al., 2017; Tiwari et al., 2018). Hepatotoxicity parameters were specifically studied using five relevant biomarkers, ALP, SGOT, SGPT, GGT, and LDH enzymes. Cardiac toxicity was predicted by the likelihood that a compound will block the hERG K^+^ channel. In addition, mutagenicity was predicted based on Ames Test, while carcinogenicity predicted the capability of the chalcone derivative to develop tumors in rat or mouse. Lastly, the acute toxicity prediction was based on the amount of orally administered chemical (in mg/kg body weight) required to kill 50% of the rats tested.

### Cytotoxicity Assay

RAW 264.7 macrophages were cultured in DMEM medium (Sigma-Aldrich, USA) supplemented with 10% fetal bovine serum at 37°C in 5% CO_2_ atmosphere. Cells were plated on 96-well microplates at a density of 10^5^ cells/well and incubated at 37°C in 5% CO_2_ for 2 h. After this period, increasing concentrations of inhibitors (0.9–4,307 μM) were added and the cultures were incubated at 37°C in 5% CO_2_ for 48 h. Subsequently, cell viability was measured by a colorimetric assay using 3-(4,5-dimethylthiazol-2-yl)-2,5-diphenyl tetrazolium bromide (MTT) (Sigma-Aldrich, USA). MTT was added to the culture medium at a final concentration of 1.0 mg/mL, and cultures were incubated at 37°C in 5% CO_2_ for 3 h. MTT was transformed by the living cells in formazan crystals that were dissolved in DMSO. The concentration of formazan was measured spectrophotometrically at 570 nm (SpectraMax M5, Molecular Devices, CA). The 50% cytotoxic concentration (CC_50_) values were determined by the nonlinear regression fits of the dose-response curves using GraphPad Prism 8.0. All measurements were performed in triplicates, twice independently.

### Activity Against *Leishmania infantum* Promastigotes

Promastigotes of *Leishmania infantum* strain MHOM/BR/1974/PP75 were cultured in Schneider's medium supplemented with 10% fetal bovine serum at 26°C. First, the selected LiARG inhibitors were diluted in culture medium at concentrations ranging from 2.9 to 1,723.0 μM. Then, late-exponential phase (after 96 h growth) *L. infantum* promastigotes, at a final density of 10^5^ parasites/mL, were incubated with previously diluted inhibitors at 26°C for 96 h. Subsequently, parasite viability was determined by a colorimetric assay using resazurin (Sigma-Aldrich, USA). Resazurin was added to the culture medium at a final concentration of 0.001% (w/v), and cultures were further incubated at 26°C for 4 h (Rólon et al., 2006). Resazurin is reduced to resorufin by living cells, and the relative concentration of resorufin/resazurin was measured spectrophotometrically at 570/600 nm (SpectraMax M5, Molecular Devices, CA), respectively. Fungizone® (2.0–650.0 nM) was used as the reference drug. The 50% inhibitory concentration (IC_50_) values were determined by the nonlinear regression fits of the dose-response curves using GraphPad Prism 8.0. All measurements were performed in triplicates, twice independently.

### Activity Against *Leishmania infantum* Amastigotes

Intracellular anti-amastigote activity was determined using the promastigote recovery assay with minor modifications (Ferreira et al., 2017). RAW 264.7 macrophages were plated on 96-well microplates at a density of 10^5^ cells/well and incubated at 37°C in 5% CO_2_ for 2 h. After this period, adherent cells were washed twice with phosphate buffered saline (PBS, pH 7.2) and infected with stationary-phase *L. infantum* promastigotes at a 10:1 parasite/macrophage ratio. After 4 h of infection at 37°C in 5% CO_2_, free parasites were removed by a wash step with PBS and infected macrophages were incubated for an additional 24 h to allow parasite differentiation into amastigotes. Then, infected cells were treated with increasing concentrations of selected LiARG inhibitors (11.0–54.0 μM) for 48 h. After this period, the culture supernatant was collected for the evaluation of NO production by the Griess reaction (Misko et al., 1993), and cells were washed with PBS. Subsequently, PBHIL medium supplemented with 5% fetal bovine serum was added and the cultures were incubated at 26°C for 72 h to recover *L. infantum* promastigotes. Only viable amastigotes can differentiate into promastigotes. Then, parasite viability was determined by a colorimetric assay using MTT (1.0 mg/mL) (Sigma-Aldrich, USA), as described earlier. Fungizone® (130–540 nM) was used as the reference drug. Results were expressed as percentages of viability in relation to the control (100% viability). The 50% inhibitory concentration (IC_50_) values were determined by the nonlinear regression fits of the dose-response curves using GraphPad Prism 8.0. All measurements were performed in triplicates, twice independently.

### Selectivity Index

The selectivity index for promastigotes and intracellular amastigotes of *L. infantum* was calculated by taking the ratio between the CC_50_ obtained for the host cell and the IC_50_ obtained for the parasite. Inhibitors displaying selectivity index > 10 were considered low cytotoxic (Katsuno et al., 2015).

### Statistical Analysis

Statistical analysis was determined based on Student *t*-test and one-way ANOVA with Tukey's comparison post-test using the GraphPad Prism 8.0 software, considering *p* < 0.05 as significant.

## Results and Discussion

### LiARG Inhibition by Chalcone Derivatives

First, we investigated the inhibitory activity of a set of thirty-six chalcone derivatives, containing different substituents on rings A and B (Ventura et al., 2015), against purified recombinant LiARG. All compounds were tested at a final concentration of 100 μM. Figure 1 shows the percentages of inhibition exhibited by the thirty-six chalcone derivatives tested against LiARG. The previously known arginase inhibitor quercetin was used as a positive control (da Silva et al., 2012). A total of 16 chalcones showed inhibitory activity against LiARG. We highlight the compounds that displayed percentages of inhibition > 50%: LC14, LC32, LC34, LC37, LC39, and LC41. Chalcones LC41, LC39, and LC32 inhibited 72.3 ± 0.3%, 71.9 ± 11.6%, and 69.5 ± 7.9% of LiARG activity, respectively, presenting an inhibitory potential greater than that of the reference inhibitor quercetin (67.1 ± 10.3%). In addition, LC14 exhibited an inhibitory activity of 65.4 ± 2.8%, while LC34 and LC37 inhibited 59.9 ± 9.2 and 58.9 ± 9.2% of LiARG activity, respectively (Figure 1).

Chen et al. (2001) showed that licochalcone A was able to inhibit *L. major* and *L. donovani* fumarate reductase, an enzyme that acts in the respiratory chain of the parasites. In addition, this compound also inhibited NADH dehydrogenase and succinate dehydrogenase of *L. major*. Moreover, the compound 2′,4′-dihydroxychalcone was reported as a potent inhibitor of glycerol-3-phosphate dehydrogenase from *L. amazonensis* (Passalacqua et al., 2015). The compounds 2′,4,4′-trihydroxy-3,3′-diprenylchalcone (bipinnatone A) and 2′,4,4′-trihydroxy-3′,5′-iprenylchalcone (bipinnatone B) proved to be potential inhibitors of the same enzyme in an *in silico* study (Ogungbe et al., 2014). Recently, da Silva et al. (2019) demonstrated that flavonoids structurally related to quercetin inhibit *Leishmania amazonensis* arginase (LaARG). Among the flavonoids tested, taxifolin showed the most effective inhibition result (88%) at 100 μM. Although chalcones and chalcone-based compounds have been described as inhibitors of other *Leishmania* enzymes, to the best of our knowledge, this is the first report on the inhibition of *Leishmania* arginase by this class of flavonoids.

Previously, a synthetic chalcone [(E)-1-(2-methoxy-4-((3-methylbut-2-en-1-yl)oxy)phenyl)-3-(4-nitrophenyl)prop-2-en-1-one] with a prenyloxy group in ring A and a nitro group in ring B demonstrated a potent inhibitory activity against *Leishmania* trypanothione reductase (Ortalli et al., 2018). Here, all chalcone derivatives containing a nitro group at position 4 of ring B were able to inhibit LiARG, especially LC39 and LC41. Indeed, the presence of a nitro group in ring B was previously correlated with a strong antileishmanial activity and selectivity (de Mello et al., 2018).

### Structural Characterization of Chalcone Derivatives Interaction With LiARG

**Figure 2:**
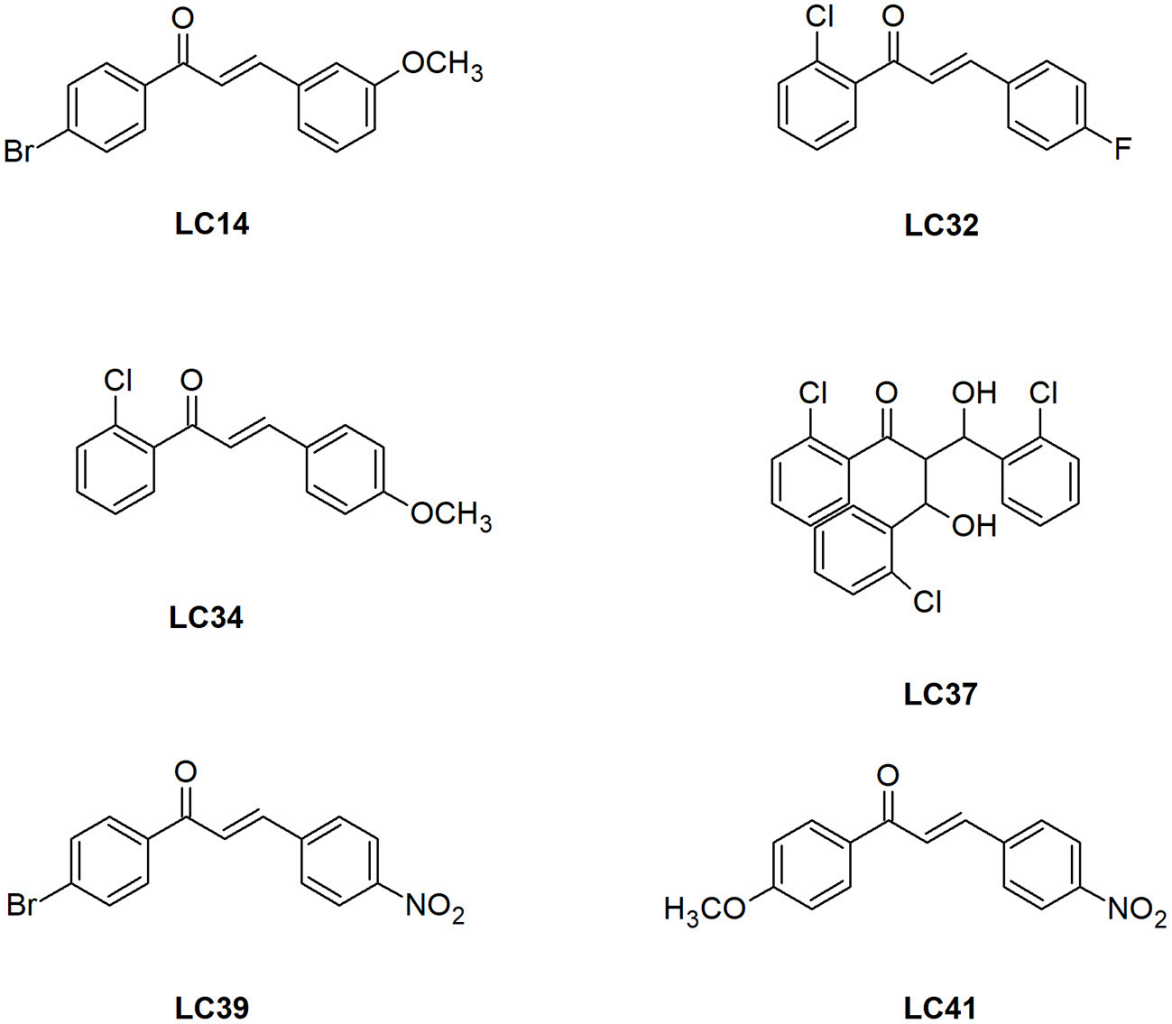
Structure of the chalcone derivatives that showed LiARG inhibition >50%. LC14: 1-(4-bromophenyl)-3-(3,4-dimethoxyphenyl)- 2-Propen-1-one; LC32: 1-(2-Chlorophenyl)- 3-(4-fluorophenyl)-2-Propen-1-one; LC34: 1-(2-Chlorophenyl)- -(4-Methoxyphenyl)-2-Propen-1-one; LC37: 3-hydroxy-2-[-hydroxy(2-Chlorophenyl)methyl]-3-(2-Chlorophenyl)-1-phenyl-1-Propanone; LC39: 1-(4-Bromophenyl)- 3-(4-Nitrophenyl)-2-Propen-1-one; LC41: 1-(4-Methoxyphenyl)- 3-(4-Nitrophenyl)-2-Propen-1-one.

To gain further insights into the mechanism of inhibition, the three most active chalcone derivatives, those with percentages of inhibition greater than that of quercetin (LC32, LC39, and LC41) (Figure 2), were docked into the active site of LiARG. For the molecular docking studies, we used the three-dimensional model of LiARG previously constructed by our group using comparative modeling (Garcia et al., 2019).

**Figure 3:**
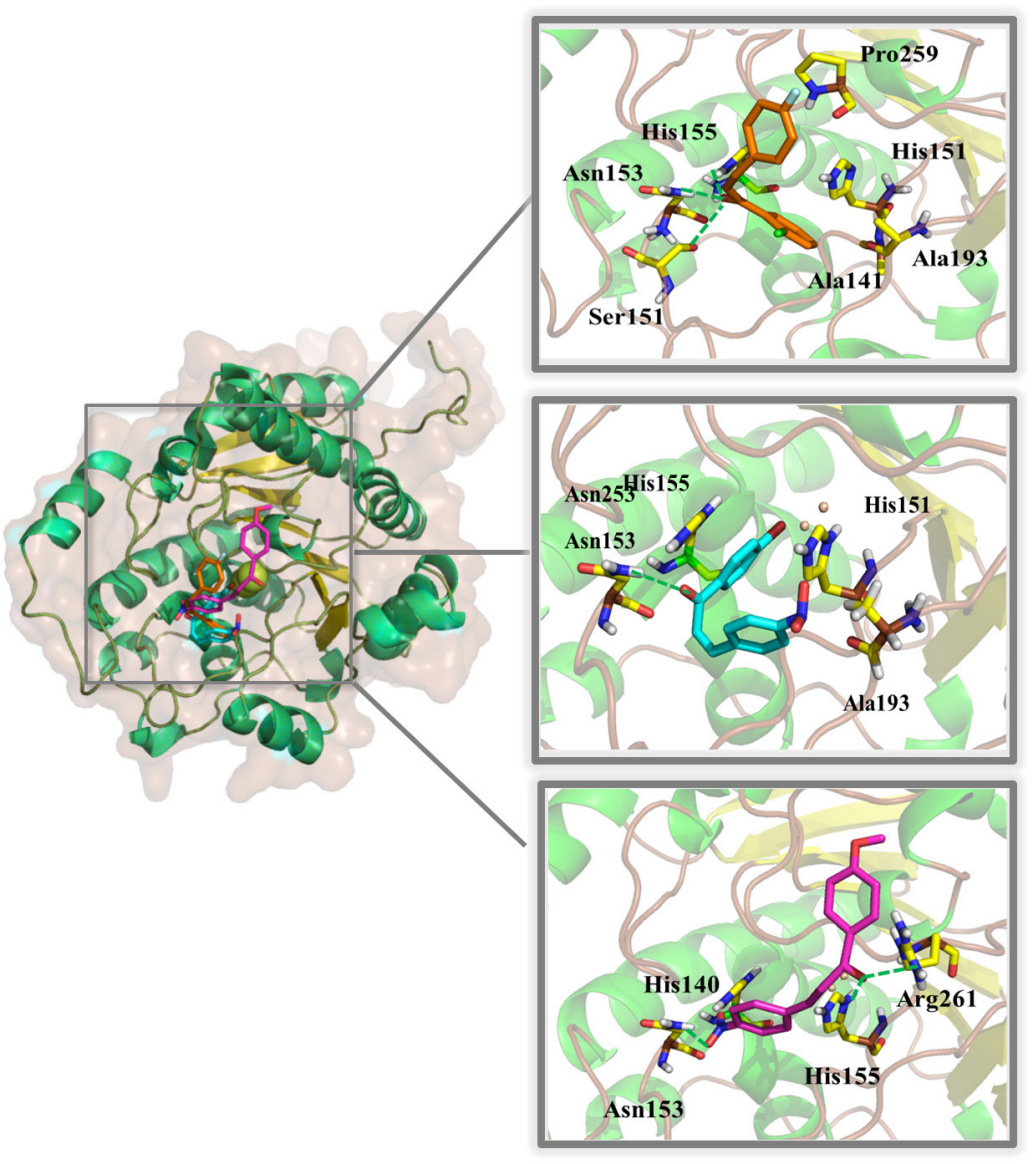
Binding mode of chalcone derivatives obtained by molecular docking. The superposition of the final docking pose of each inhibitor docked into the active site of LiARG is shown in the left. The three-dimensional model of LiARG is displayed in cartoon representation. α-Helices are colored green, β-sheets are colored yellow, and loops are colored gray. The molecular structures of chalcone derivatives are shown in sticks representation and colored accordingly: orange (LC32), cyan (LC39), and magenta (LC41). LiARG amino acid residues that directly engage in inhibitor binding are marked yellow and labeled. Hydrogen bonds are shown as dashed lines and colored green.

The binding modes of LC32, LC39, and LC41 showed estimated binding energy values of −4.42, −6.91, and −3.80 kcal/mol, respectively (Figure 3). The docking results suggested that LC32 interacts with the active site of LiARG through three possible hydrogen bonds between the oxygen atom of the compound's carbonyl group and the polar side chains of residues Ser151 (OH—O distance of 2.8Å), Asn153 (NH—O distance of 1.9Å) and His155 (NH—O distance of 2.1Å). Moreover, the 2-clorophenyl group makes van der Waals and hydrophobic interactions with His140, Ala 141, Ser151, Ala193, and Pro259 (Figure 3). In contrast, LC39 makes one hydrogen bond between its carbonyl group and the nitrogen atom of Asn153 side chain (NH—O distance of 1.9Å). In addition, LC39 interacted mainly by van der Waals and hydrophobic contacts with residues His140, His155, and Ala193 (Figure 3). It is worth noting that His140, Asn153, and His155 are conserved among all arginases and have been described as key residues for ligand binding with *L. mexicana* arginase (Ash, 2004; D'Antonio et al., 2013). Lastly, three hydrogen bonds were observed for the LC41:LiARG complex. Two of them are between the compound's carbonyl group and the oxygen atom of the side chains of His155 (NH—O distance of 2.1Å) and Arg261 (NH—O distance of 2.5Å), while the third occurs between the hydroxy-imino-λ1–oxidanyl-phenyl group and the amidic nitrogen of Asn153 side chain (NH—O distance of 1.9Å). LC41 also has van der Waals interactions with His140 (Figure 3). Interestingly, residues Ala141, Ser151, and Ala193 occupy the same positions as Ala140, Ser150, and Ala192, respectively, in *L. mexicana* arginase, where they interact with the nor-NOHA, ABH, and BEC competitive inhibitors (da Silva et al., 2012; D'Antonio et al., 2013; Hai and Christianson, 2016). Moreover, the observed interaction between LC32 and Ala141 was also described for the rosmarinic acid:LiARG complex by molecular docking (Garcia et al., 2019). Remarkably, the chalcone derivatives exhibited more van der Waals and hydrophobic contacts with LiARG than rosmarinic acid. Glisic et al. (2016) prospected 5667 flavonoids in the MetIDB database using an EIIP/AQVN filter and 3D QSAR. Ten compounds were selected for docking into the *Leishmania* arginase model structure, and compound 39 was selected as the best inhibitor. This compound displayed interactions with Ser150(H), Asn143(H), Asp194(H), Ala192(A), Thr257(A) residues (Glisic et al., 2016). Taken together, the results suggested that flavonoid scaffolds may be starting points for drug design aiming Leishmania ARG inhibition and disease control.

### Computational Pharmacokinetic and Toxicological Evaluations of Chalcone Derivatives

In the drug design context, pharmacokinetic and toxicological properties are crucial aspects to achieve good oral bioavailability and safe drugs. Thus, the ADMET computational evaluations were carried out to compare the three chalcone derivatives with the greatest inhibitory activity against LiARG (LC32, LC39, and LC41) (Figure 2) with miltefosine, the only oral treatment available for leishmaniasis. Our results suggest that these derivatives exhibit good oral bioavailability, based on the Lipinski rule of 5 (Lipinski, 2004). In addition, LC32, LC39, and LC41 showed inhibitory potency against CYP1A2 and 3A4 and a potential to be substrates for CYP1A2, CYP2B6, CYP2C9, CYP2D6, CYP2E1, and CYP3A4 isoforms. CYP3A4 is one of the main isoforms of cytochrome P450, responsible for xenobiotic metabolism. Overall, its inhibition may cause drug-drug interactions (Tirona and Kim, 2017) (Table 1).

**Table 1:** *In silico* pharmacokinetic and toxicological properties of the most active chalcones derivatives against LiARG (LC32, LC39, and LC41) and miltefosine.

**Drug/Chalcones**	**ADMET predictor**							
	**Pharmacokinetic properties**			**Toxicological endpoints**				
	**CYP inhibition**	**CYP substrate**	**Rule of 5**	**hERG inhibitor**	**Hepatotoxic**	**Mutagenic**	**Carcinogenic**	**Acute toxicity in rats**
LC32	1A2, 2C19 3A4	1A2, 2A6, 2B6, 2C8, 2C9, 2C19, 2D6, 3A4	Yes	Yes	No	No	No^R,M^	726.79
LC39	1A2, 2C19, 3A4-	1A2, 2A6, 2B6, 2C9, 2C19, 2D6, 2E1. 3A4	Yes	No	No	Yes	No^R,M^	1,568.17
LC41	1A2,3A4	1A2,2A6, 2B6, 2C9, 2D6, 2E1, 3A4	Yes	No	No	Yes	No^R,M^	2,374.28
Miltefosine	–	–	Yes	No	No	No	No^R,M^	855.10

*R: rats; M: mice*.

Concerning the toxicity endpoints evaluated, only LC32 showed potential to act as a hERG inhibitor, and thus we predicted cardiotoxicity. Fortunately, LC32, LC39, LC41, and miltefosine displayed no risks of hepatotoxicity, carcinogenicity and acute rat toxicity (Table 1). These *in silico* results support the findings of de Mello et al. (2018) that chalcones with activity against *Leishmania* generally show good pharmacokinetic and low toxicity profiles (de Mello et al., 2018). Therefore, the chalcone derivatives LC32, LC39, and LC41 may support the research and development of more effective and less toxic LiARG inhibitors.

### Antileishmanial and Cytotoxic Activities of Chalcone Derivatives

After this first screening, the chalcone derivatives exhibiting more than 50% LiARG inhibition (LC14, LC32, LC34, LC37, LC39, and LC41) (Figure 2) were selected for the investigation of their activity against the promastigote forms of *L. infantum*. Fungizone® was used as a reference drug control. The results demonstrated in Table 2 reveal that all selected chalcone derivatives displayed antileishmanial activity, albeit with IC_50_ values higher than that of Fungizone® (IC_50_ of 23.8 ± 0.7 nM). LC32 exhibited the greatest effect against *L. infantum* promastigotes with an IC_50_ of 74.1 ± 10.9 μM. Then, chalcones LC14, LC41, and LC39 showed similar IC_50_ values of 283.4 ± 14.2, 319.1 ± 14.3, and 398.0 ± 44.2 μM, respectively. Lastly, LC34 showed an IC_50_ of 747.2 ± 22.3 μM, and LC37 was the only chalcone derivative that did not display *in vitro* activity against *L. infantum* promastigotes at the highest concentration tested.

**Table 2:** LiARG inhibition, anti-*L. infantum* activity and cytotoxicity of chalcone derivatives.

**Chalcone**	**ARGLi inhibition (%)** **(100 μM)**	**RAW 264.7**	**Promastigotes**	**Amastigotes**	**Selectivity Index**	
		**CC_**50**_ ± SE** **(μM)**	**IC_**50**_ ± SE** **(μM)**	**IC_**50**_ ± SE** **(μM)**	**PRO**	**AMA**
LC14	65.4 ± 2.8	75.1 ± 8.9	283.4 ± 14.2	n.d.	<1	n.d
LC32	69.5 ± 7.9	479.1 ± 19.5	74.1 ± 10.9	111.5 ± 19.8	6.5	4.3
LC34	59.9 ± 9.2	3,010.9 ± 88.0	747.2 ± 22.3	65.4 ± 10.9	4.0	46.0
LC37	58.9 ± 9.2	>4,000	>1,500	n.d.	n.d.	n.d
LC39	71.9 ± 11.6	4531.0 ± 212.0	398.0 ± 44.2	42.3 ± 17.1	11.4	107.1
LC41	72.3 ± 0.3	1,146.8 ± 58.8	319.1 ± 14.3	43.7 ± 13.7	3.6	26.2
Fungizone®	n.d	11.9 ± 0.2	0.024 ± 0.0007	0.138 ± 0.01	503.2	86.9

Next, we evaluated the cytotoxicity of the selected chalcone derivatives against RAW 264.7 macrophages, enabling us to determine their selectivity against parasite cells. LC39 showed a CC_50_ of 4,531.0 ± 212.0 μM and thus a SI of 11.4, proving to be more than 10 times selective against *L. infantum* promastigotes. In contrast, LC14 displayed a CC_50_ of 75.1 ± 8.9 μM (SI < 1), being more toxic to the host cell than the parasite. The chalcone derivatives LC34, LC41, and LC32 showed CC_50_ values of 3,010.9 ± 88.0 (SI of 4.0), 1,146.8 ± 58.8 (SI of 2.6), and 479.1 ± 19.5 μM (SI of 6.5), respectively. Indeed, the replacement of a bromide at position 4 of ring A of LC39 by a methoxy group in LC41 may play an important role in the cytotoxicity for macrophages. In addition, LC37, which did not show anti-*L. infantum* promastigote activity, also did not inhibit the growth of RAW 264.7 macrophages at the highest concentration tested.

Considering their anti-*L. infantum* promastigote activity and selectivity against parasite cells (SI > 1.0), the chalcone derivatives LC32, LC34, LC39, and LC41 were selected for the analysis of their activity against *L. infantum* intracellular amastigotes. Fungizone® was again used as a reference drug control. *L. infantum*-infected peritoneal macrophages were treated with the selected chalcone derivatives and the viability of promastigotes recovered from infected macrophages was measured. All chalcone derivatives were able to reduce the parasite load when compared to untreated control cells, displaying IC_50_ values of 42.3 ± 17.1 (LC39), 43.7 ± 13.7 (LC41), 65.4 ± 10.9 (LC34), and 111.5 ± 19.8 μM (LC32) (Table 2). Maquiaveli et al. (2017) demonstrated that ARG inhibitors can interact with their target in infected macrophages. The authors reported that verbascoside, a naturally occurring caffeoyl phenylethanoid glycoside, decreased the number of intracellular amastigote forms of *L. amazonensis* by arginase inhibition. Indeed, they observed that the anti-amastigote activity of verbascoside was reversed by the addition of l-ornithin, suggesting that inhibition of parasite ARG is the most likely mode of action. Here, we provide important evidence that compounds LC32, LC34, LC39, and LC41 hamper parasite burden in macrophages by ARG inhibition. However, further investigation is still necessary in order to confirm ARG as a target in the cellular model.

Chalcone LC39 showed the lowest IC_50_ value against intracellular amastigotes, resulting in a SI of 107.1, even greater than that of the reference drug Fungizone® (IC_50_ of 0.138 ± 0.01 μM; SI of 86.9). It is noteworthy that a SI over 10 is considered ideal according to the hit and lead criteria in drug discovery for infectious diseases previously established by Katsuno et al. (2015). Indeed, the presence of a nitro group in ring B seems to influence the antileishmanial activity, as suggested by Gomes et al. (2017a). The authors showed that the presence of a 5-nitrofuran group in ring B enhanced the bioactivity against *L. infantum*. In this study, the elevated SI value indicates LC39 as a promising antileishmanial agent, as selectivity is a highly desired feature in leishmanicidal drugs.

The relationship between the chalcone's structure and its antileishmanial activity was recently reviewed by Tajuddeen et al. (2018). The authors reported that aryl rings, which constitute the core pharmacophores of chalcones, are essential for the bioactivity. In fact, the presence of bulky substituents at positions 2′ and 3′ of ring B seems to increase chalcone activity, while the same substituents at position 4′ leads to a decrease in the antileishmanial effect. However, alpha propane chain α-β unsaturated seems to have low influence on the antileishmanial activity of chalcones. Interestingly, LC37, which presents substitutions in the propane chain, inhibited LiARG but displayed neither antileishmanial nor cytotoxic effects against macrophages at the highest concentrations tested.

**Figure 4:**
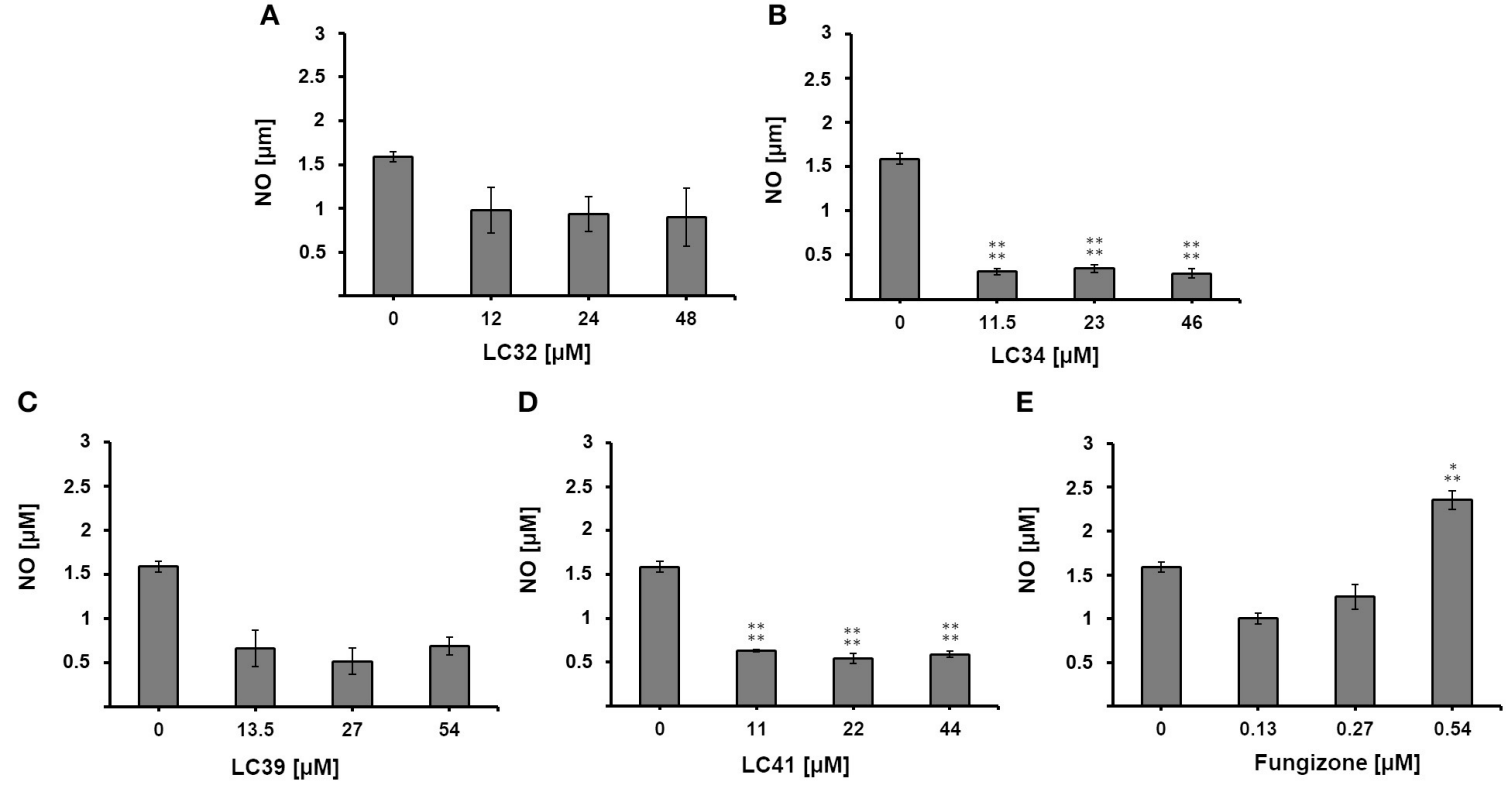
Effect of chalcone derivatives on the production of nitric oxide by *L. infantum*-infected macrophages. *L. infantum*-infected macrophages were treated with increasing concentrations of **(A–D)** chalcone derivatives (11–54 μM) or **(E)** the reference drug fungizone (0.13–0.54 μM) and nitrite concentration was measured by the Griess reaction. Control represents the amount of nitric oxide produced by *L. infantum*-infected macrophages in the absence of any treatment. Statistical analysis of the differences between mean values obtained for the experimental groups was done by ANOVA with Tukey's *post hoc* test. *P*-value < 0.0005 (three asterisks), *p* < 0.0001 (four asterisks) were considered significantly different from the control.

To further investigate the nature of the antileishmanial activity, we evaluated whether treatment with the selected chalcone derivatives (LC32, LC34, LC39, and LC41) increased NO production by *L. infantum*-infected macrophages. For this, the supernatant of infected and treated macrophages was analyzed by the Griess reaction. *In vitro* treatment with LC32 and LC41 significantly decreased the production of NO by *L. infantum*-infected macrophages when compared to the untreated control cells. In addition, LC34 and LC39 showed no significant difference to the untreated control (Figure 4). The results presented here corroborates those reported by Ventura et al. (2015), which showed the inhibition of NO production by RAW 264.7 macrophages after treatment with LC14 and LC41 (IC_50_ for NO of 58.9 ± 6.8 and 13.5 ± 5.5 μM, respectively). Indeed, previous studies have already connected the anti-inflammatory effect of chalcones to the inhibition of NO production (Ban et al., 2004; Park et al., 2009a,b; Reddy et al., 2011; Kim et al., 2013). Our results suggest that the chalcone derivatives were unable to modulate the host immune response and thus their antileishmanial activity may occur in a nitrosative stress-independent fashion, most likely directly on the parasite by inhibiting *Leishmania* arginase. ARG inhibition may lead to polyamine depletion, which may consequently affect parasite metabolism and proliferation. In addition, the enzyme inhibition could compromise the parasite redox balance, since the lack of spermidine impairs trypanothione production (Muxel et al., 2018). As a result, parasites would be more susceptible to oxygen reactive species generated by the host cell defense. Interestingly, other consequences of arginase inhibition have been suggested. Recently, transcriptomic data comparing *Leishmania amazonensis arg*^−^ to parasite wild-type (WT) revealed that arginase downregulates several virulence factors, including LPG, PPG, GP63, and amastin (Aoki et al., 2019).

## Conclusion

We report for the first time on the ability of chalcones to inhibit *L. infantum* arginase. Among the chalcone derivatives tested against LiARG, three (LC32, LC39, and LC41) showed inhibitory potential greater than the reference inhibitor quercetin. *In silico* studies indicated the direct interaction of chalcone derivatives with LiARG's active site residues as well as their low toxicity and good oral bioavailability. In addition, the chalcone derivatives LC34, LC39, and LC41 were effective against the promastigote and intracellular amastigote forms of *L. infantum*. Remarkably, LC39 stood out for being highly selective to the parasite, even more so than the reference drug Fungizone®. Interestingly, our results point at the nitro group substituent in ring B as an important factor for the antileishmanial effect of chalcones, corroborating previous findings. Taken together, the results presented here bring new perspectives for *Leishmania* arginase inhibitors and the development of chalcone-based drug candidates against visceral leishmaniasis.

## Data Availability Statement

The raw data supporting the conclusions of this article will be made available by the authors, without undue reservation.

## Author Contributions

AV, AP, and IR designed the work. AG, JJ, AP, and IR wrote the manuscript. AG and DO performed the enzymatic assays and data analysis. AMTS, and ARS performed the *in silico* analysis. IL, RS, and LM synthesized the chalcone derivatives. AG performed the biological assays. All authors contributed to the article and approved the submitted version.

## Conflict of Interest

The authors declare that the research was conducted in the absence of any commercial or financial relationships that could be construed as a potential conflict of interest.
